# Mucosal-associated invariant T (MAIT) cell responses in *Salmonella enterica* serovar Typhi strain Ty21a oral vaccine recipients.

**DOI:** 10.1093/oxfimm/iqaf002

**Published:** 2025-03-25

**Authors:** Shubhanshi Trivedi, Olivia J Cheng, Ben J Brintz, Richelle C Charles, Daniel T Leung

**Affiliations:** Division of Infectious Diseases, Department of Internal Medicine, University of Utah, Salt Lake City, UT, 84132, United States; Division of Infectious Diseases, Department of Internal Medicine, University of Utah, Salt Lake City, UT, 84132, United States; Division of Infectious Diseases, Department of Internal Medicine, University of Utah, Salt Lake City, UT, 84132, United States; Division of Epidemiology, Department of Internal Medicine, University of Utah, Salt Lake City, UT, 84108, United States; Division of Infectious Diseases, Department of Medicine, Massachusetts General Hospital, Boston, MA, 02114, United States; Department of Medicine, Harvard Medical School, Boston, MA, 02115, United States; Department of Immunology and Infectious Diseases, Harvard T.H. Chan School of Public Health, Boston, MA, 02115, United States; Division of Infectious Diseases, Department of Internal Medicine, University of Utah, Salt Lake City, UT, 84132, United States; Division of Microbiology and Immunology, Department of Pathology, University of Utah, Salt Lake City, UT, 84132, United States

**Keywords:** Mucosal-associated invariant T (MAIT) cells, *Salmonella enterica* serovar Typhi, typhoid fever, mucosal tissue, vaccine, vaccination, chemokine receptors

## Abstract

Mucosal–associated invariant T (MAIT) cells are unconventional innate-like T cells abundant in human mucosal tissues and are associated with protective responses to microbial infections. MAIT cells have the capacity for rapid effector functions, including the secretion of cytokines and cytotoxic molecules. In this study, we examined the longitudinal circulating MAIT cell response to the live attenuated oral vaccine Ty21a (Ty21a) against *Salmonella enterica* serovar Typhi (*S*. Typhi). We enrolled healthy adults who received a course of oral live-attenuated *S.* Typhi strain Ty21a vaccine and assessed peripheral blood MAIT cell longitudinal responses pre-vaccination, and at seven days and one-month post-vaccination, using flow cytometry, cell migration, and tetramer decay assays. We showed that following vaccination, circulating MAIT cells were lower in frequency, but were more activated, and had higher levels of gut-homing marker integrin α4β7 and chemokine receptors CCR9 and CCR6, suggesting the potential of MAIT cells to migrate to mucosal sites. We found no significant differences in MAIT cell functionality, cytotoxicity and T-cell receptor avidity, except in TNF expression, which was higher post-vaccination. We show that MAIT cell immune responses are modulated post-vaccination against *S.* Typhi. This study contributes to our understanding of MAIT cells’ potential role in oral vaccination against bacterial mucosal pathogens.

## Introduction

Mucosal-associated invariant T (MAIT) cells are innate-like αβ T cells defined by the expression of an invariant α chain, generally Vα7.2 linked to Jα33, Jα12, or Jα20, and a limited repertoire of T cell receptor β (TCRβ) chains in humans [1, 2]. MAIT cells are restricted by the non-classical MHC-related molecule 1 (MR1) and respond to vitamin B metabolites derived from bacterial and fungal species [3]. MAIT cells have been associated with protection and antibacterial immune defense in various bacterial infections, including *Legionella longbeachae*, *Mycobacterium tuberculosis*, *Mycobacterium bovis*, *Francisella tularensis*, *Escherichia coli*, *Vibrio cholerae,* and *Klebsiella pneumoniae* [4–8]. Exposure to enteric bacteria in clinical studies, such as in challenge studies with *S*. Typhi and *S*. Paratyphi A [9, 10], and in vaccine trials with *Shigella flexneri* [11], MAIT cell activation, proliferation, and homing to mucosal sites were observed. MAIT cell functions include the capacity to secrete TNF, IFN-γ, IL-17, as well as Granzyme B upon activation [12, 13]. MAIT cells are potential targets for improving vaccine responses [14] as they bridge the adaptive and innate immune responses against bacterial infections and are donor-unrestricted (not restricted by MHC polymorphism) [15].

Emerging evidence supports a role of MAIT cells in the response to vaccination [9, 10, 16, 17]. In participants vaccinated with an attenuated strain of *Shigella dysenteriae*, MAIT cell activation was observed in those who had mounted LPS-specific IgA antibody-secreting cell responses [11]. In bacterial challenge studies, MAIT cells are activated and decline in blood upon human challenge with *S.* Typhi [10]. A recent study revealed that MAIT cells play a crucial role in the initial priming of adaptive T cell immune responses to antigens encoded by the ChAdOx1 viral vaccine vector in both mice and humans [17]. However, it remains unclear whether this finding extends to other vaccine modalities such as the orally administered attenuated *S.* Typhi strain Ty21a, a commercially available vaccine for typhoid fever [18]. Therefore, the objective of our study was to examine the longitudinal circulating MAIT cell responses in a cohort of Ty21a recipients. We showed that Ty21a vaccination resulted in changes in MAIT cell frequency, activation, cytokine production and homing markers expression.

## Materials and methods

### Subjects

Eleven healthy participants took part in this study, including six males (aged 42 ± 15 years) and five females (aged 40 ± 10 years). All participants provided written informed consent. Four doses of a single oral capsule of Ty21a (Vivotif, PaxVax) were taken on day 1, 3, 5 and 7. Blood samples were collected from each subject at study enrollment (day 0), and approximately 7 days and 1 month after the last dose of vaccine (day 7- and 1-month post-vaccination, p.v.). Venous blood was collected and centrifuged over a Lymphoprep (STEMCELL Technologies Inc) density gradient using a standard protocol to isolate peripheral blood mononuclear cells (PBMCs). PBMCs in cryogenic vials were placed immediately into an isopropanol freezing container (Nalgene Mr Frosty) and were cryopreserved at −80°C until used for immunologic assays. Study procedures were reviewed and approved by the Institutional Review Board of the University of Utah (IRB #84287).

### Antigenic stimulation and incubation

Viable *S.* Typhi Ty21a were obtained by dissolving a vaccine capsule (Vivotif) in 10 ml brain heart infusion (BHI) media and incubating overnight at 37°C. The bacteria were then sub cultured (1 : 10) in BHI media for 4 h (O.D = 0.4), harvested and stored in 50% glycerol at −80°C. For phenotypic analysis of MAIT cells, PBMCs were thawed from −80°C and were seeded (2 × 10^6^ cells/well) in complete medium in 96-well U bottom plates. Cells in each well were stimulated at a multiplicity of infection (MOI) of 100 with heat-killed *S.* Typhi Ty21a (killed by incubation at 95°C for 30 min) to mimic the immune response of MAIT cells *in vivo*, consistent with a prior study that utilized similar methods and elicited MAIT responses in Ty21a vaccinees [19]. Unstimulated control wells were treated with complete medium (RPMI 1640 with 10% FBS, 1% penicillin/streptomycin, and 15 mM HEPES). Cells were then incubated at 37° in 5% CO_2_. After an overnight incubation, brefeldin A (3 μg/ml) (BD GolgiPlug; BD Biosciences) was added to each well, and the plate was incubated for a further 4 h at 37° in 5% CO_2_ prior to cell staining for flow cytometry_._ In pilot experiments, we used fixed *E. coli* stimulation of PBMCs as a positive control [20, 21]. However, due to limitations in the number of PBMCs available from each subject, we did not use positive controls for subsequent experiments.

### Flow cytometry

Prior to surface staining, PBMCs were labeled with Zombie UV Fixable Viability Dye (BioLegend). The cells were then washed and incubated with fluorochrome-conjugated antibodies and the 5-OP-RU tetramer (NIH Tetramer Core) for 40 min at room temperature (RT). Before surface staining, the cells were incubated in a human Fc block (BD) for 15 min at RT [22]. Details of viability dye and antibodies used are listed in Supplementary Table 1. For intracellular cytokine analysis, surface staining was followed by fixation, permeabilization, and staining using the Foxp3/transcription factor staining buffer set (eBioscience). Compensation beads (BD Biosciences) were used to create compensation matrices, and Fluorescence minus One (FMO) control were used to identify populations of interest. We defined MAIT cells as live CD45^+^ CD19^−^ TCRγδ^−^ CD4^−^ CD3^+^ CD8^+^ CD161^+^ Vα7.2^+^ MR1-5-OP-RU tetramer^+^ (Figs 2–4). No significant differences were observed in the frequencies of CD4+ Vα7.2^+^ MR1-5-OP-RU tetramer^+^ MAIT and CD4^+^ CD8^+^ Vα7.2^+^ MR1-5-OP-RU tetramer^+^ MAIT cells in post-vaccination samples compared to pre-vaccination samples (supplementary figure 2G and H). A total of 10^6^ events per sample were acquired using Cytek Aurora (Cytek Biosciences) and analyzed using FlowJo software v10 (Tree Star Inc, Ashland, OR).

### Cell migration assay

To assess migratory properties of MAIT cells, PBMCs were stimulated with heat-killed *S.* Typhi Ty21a overnight, washed, and resuspended in RPMI 1640 medium supplemented with 0.1% bovine serum albumin (Thermo Fisher Scientific, MA, USA). Cells were seeded in the upper chamber of a 6-well transwell plate inserted with a pore size of 3 μm (Thermo Fisher Scientific, MA, USA) at a density of 1 × 10^6^ cells/well. Cells were allowed to migrate against a gradient of 150 ng/ml recombinant chemokines CCL20/MIP-3α and CCL25/TECK-3 (Peprotech, NJ, USA) in RPMI 1640 medium supplemented with 0.1% bovine serum albumin for 4 h at 37°C [23]. Cells migrated into the bottom chamber were collected, washed in FACS buffer (phosphate-buffered saline with 2% fetal bovine serum), stained with fluorochrome-conjugated anti-CD8a, anti-TCRVα7.2, anti-CD161 antibodies and anti-human MR1 5-OP-RU Tetramer (NIH Tetramer Core Facility), and MAIT cells were counted using Countbright^TM^ absolute counting beads (Thermo Fisher Scientific, MA, USA) and flow cytometry.

### Tetramer staining and decay assay

Tetramer staining and decay assay were performed as previously described [9]. Briefly, PBMCs were stained with live/dead fixable viability dye efluor 780 (eBiosciences, Thermo Fisher Scientific, MA, USA) for 15 min at room temperature (RT), followed by an incubation with 5-OP-RU MR1 tetramer for 40 min at RT. Excess tetramer was washed off and cells were re-suspended in FACS buffer with or without 20 μg/ml anti-MR1 antibody (clone 26.5, Biolegend). Samples were left at 37°C and periodic samples were taken, washed and fixed immediately. CD3 staining was performed after collecting all samples and cells were analyzed using flow-cytometry.

### ELISAs

For enzyme-linked immunosorbent assays (ELISA), we used *S*. Typhi LPS antigen and assessed plasma antibody responses (immunoglobulin [Ig] M, IgA, and IgG). ELISAs were performed as previously described [24]. Briefly, microplates (nunc-maxisorp flat-bottom 96-well plates, Invitrogen) were coated with 1 μg/ml LPS, and plasma was added at a dilution of 1 : 200 for IgG and 1 : 100 for IgA and IgM. Bound antibodies were detected with anti-human IgG, IgA, and IgM conjugated with horseradish peroxidase (Jackson ImmunoResearch), and plates were developed by adding 100 μl/well TRB substrate for 10 min in the dark. Development was stopped by the addition of 100 μl/well of 0.2 N H_2_SO_4_ and OD read at 450 nm in an ELISA microplate reader (Multiskan Ascent; Thermo Labsystems).

### Statistical analysis

Paired comparisons were performed using Wilcoxon matched-pairs signed-rank test using Prism v9 (GraphPad). P values are two-tailed and considered significant at *P* < .05.

## Results

### Ty21a vaccination resulted in a decrease in frequency of circulating MAIT cells but an increase in activation

**Figure 1.**
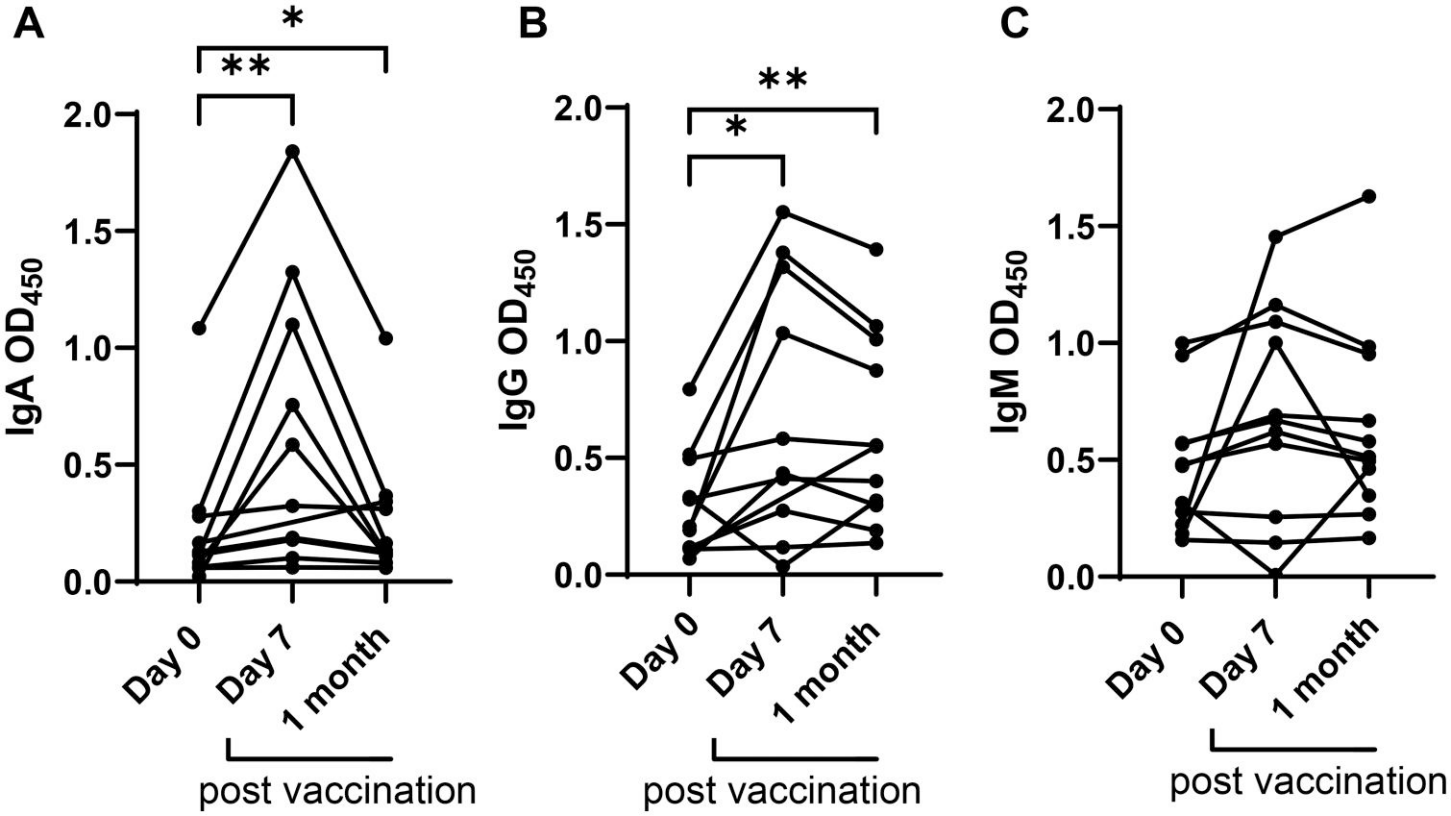
Changes in *S*. Typhi specific antibody response post-vaccination. Plasma ELISA’s against *S*. Typhi LPS (A–C) IgA, IgG and IgM antibody responses pre-vaccination (day 0) and post-vaccination (day 7 and 1 month). The data are representative of two independent experiments out of a total of two. *P < .05, **P < .01, in Wilcoxon signed-rank test (paired samples).

Analysis of plasma antibody responses to *S.* Typhi LPS in Ty21a vaccine recipients revealed that vaccine elicited significantly higher IgA and IgG antibody responses at 7 days and 1-month post-vaccination compared to day 0 (pre-vaccination) (Fig. 1A and B). No significant differences were found in IgM antibody responses (Fig. 1C). These results are consistent with prior studies of Ty21a vaccination [25–27].

**Figure 2.**
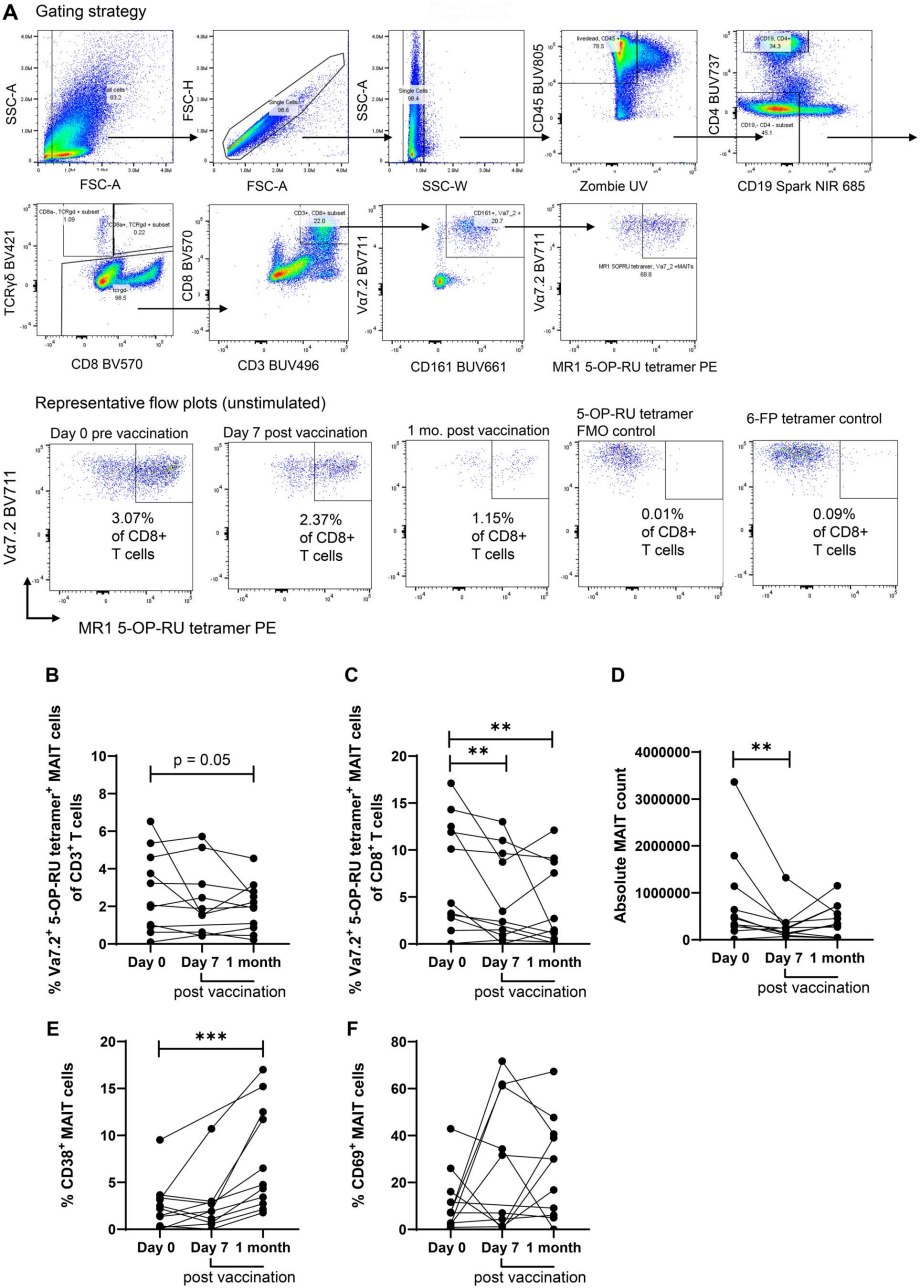
MAIT cell frequency and activation post-vaccination. (A) MAIT cell gating strategy and representative flow cytometry plots from unstimulated samples. (B) MAIT cells frequency as percentage of CD^3+^ T cells and (C) as percentage of CD^8+^ T cells. (D) absolute MAIT cell counts (CD^3+^ MAIT) pre-vaccination and post-vaccination. (E and F) Frequency of activated MAIT cells expressing CD38 and CD69 pre- and post-vaccination. Black circles are unstimulated donors. The data are representative of two independent experiments out of a total of two. *P <0.05, **P <0.01, ***P <0.001 in Wilcoxon signed-rank test (paired samples).

Because of the potential importance of MAIT cells against *S.* Typhi infections and as potential targets for vaccine development, we assessed whether immunization with the Ty21a typhoid vaccine elicits MAIT cell immune responses. PBMCs from Ty21a vaccinees were isolated and stimulated with heat-killed *S.* Typhi Ty21a, and MAIT cell frequency and phenotype were analyzed using flow cytometry. We defined MAIT cells as live CD45^+^ CD19^−^ TCRγδ^−^ CD4^−^ CD3^+^ CD8^+^ CD161^+^ Vα7.2^+^ MR1-5-OP-RU tetramer^+^, and we used fluorescence minus one (FMO) controls of the MR1-5-OP-RU tetramer and the 6-FP-tetramer for gating MAIT cells (Fig. 2A). We found that the frequencies of circulating MAIT cells as a proportion of total CD3^+^ T cells and as a proportion of total CD8^+^ cells significantly decreased at day 7 and at one-month post-vaccination compared to pre-vaccination (Fig. 2B and C). Absolute MAIT cell counts were also lower both at day 7 and at one-month post-vaccination compared to pre-vaccination (Fig. 2D and Supplementary Table 2).

We next measured the expression of activation markers CD38 and CD69 in MAIT cells. We found that the frequencies of CD38^+^ MAIT cells (Fig. 2E) increased at one-month post-vaccination (CD38 positive population of Vα7.2^+^ CD161+ cells were also higher at one-month post-vaccination compared to pre-vaccination (Supplementary Fig. 1)). No significant differences between post-vaccination and pre-vaccination were found in CD69^+^ MAIT cells (Fig. 2F). Conventional CD4^+^ and CD8^+^ T cells frequencies increased post-vaccination, consistent with a previous publication, [28] and the frequencies of CD14^+^ monocytes decreased in circulation post-vaccination (Supplementary Fig. 2). No significant differences were found in B cells, CD4^+^ CD8+ MAIT cells, CD4^+^ MAIT cells and TCRγδ cells at the time points assessed post-vaccination compared to pre vaccination (Supplementary Fig. 2). Taken together, we found that vaccination with Ty21a impacted circulating MAIT cell frequency and activation.

### Ty21a vaccination resulted in higher frequencies of TNF expressing circulating MAIT cells

**Figure 3.**
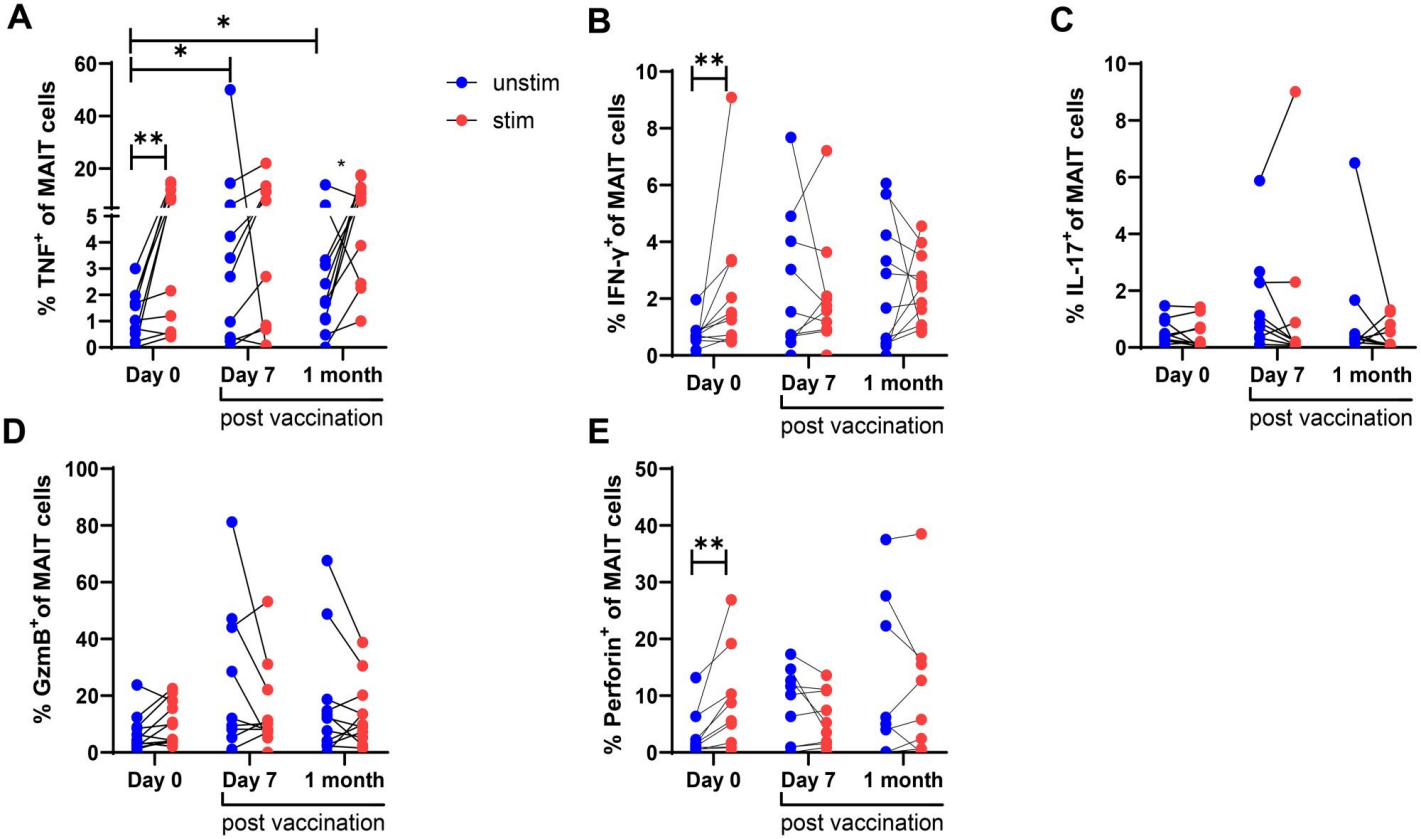
MAIT cells effector function post-vaccination. PBMCs obtained from vaccine recipients (*n* = 11 per group) were stimulated with heat-killed *S.* Typhi at MOI of 100 and intracellular expression of (A) TNF (B) IFN-γ, (C) IL-17, (D) granzyme B and (E) Perforin in MAIT cells (CD^3+^ 5-OP-RU tetramer+ CD8+) were analyzed using flow cytometry (blue circle shows unstimulated and red circle shows stimulated data). Data were expressed as mean ± SEM. The data are representative of two independent experiments out of a total of two. Paired comparisons were made using Wilcoxon matched-pairs signed-rank test using Prism v9 (GraphPad). *P < .05, **P < .01, ***P < .001.

We next examined whether vaccination with Ty21a induces MAIT cells to express effector molecules such as IFN-γ, TNF, IL-17a and cytotoxic molecules. Using flow cytometry, we found a significant increase in the frequencies of MAIT cells intrinsically expressing TNF at day 7- and 1-month post-vaccination compared to pre-vaccination, only in the unstimulated condition (Fig. 3A). This indicates that the intrinsic TNF responses in MAIT cells increased following vaccination. IFN-γ expressing MAIT cells also were non-significantly higher (*P* = .08) post-vaccination in unstimulated conditions (Fig. 3B and Supplementary Fig. 3). No significant differences were found in IL-17a, granzyme B, or perforin expression among the groups (Fig. 3C–E). IFN-γ, TNF and perforin expressing MAIT cells significantly increased in response to *ex-vivo S.*Typhi stimulation compared to unstimulated condition at the pre-vaccination timepoint. However, except for TNF at 1-month post-vaccination, we did not observe any significant differences between unstimulated and stimulated conditions at any of the post-vaccination timepoints (Fig. 3A).

To determine whether vaccination changes the TCR avidity of the circulating MAIT cell population for 5-OP-RU–MR1 complexes, we stained PBMCs with 5-OP-RU tetramers, and assayed the rate of tetramer dissociation by incubating cells over a time course with an anti–MR1 blocking antibody [9]. We hypothesized that vaccination could selectively enhance or activate a subset of high-avidity MAIT cells. Despite the observed decrease in MAIT cell numbers post-vaccination, an increase in avidity would suggest preferential survival or expansion of high-avidity clones. In the presence of anti-MR1 antibody, tetramer dissociated rapidly over an hour interval in both pre- and post-vaccination groups. Using the MFI of MAIT cells bound to tetramer and a two-phase (fast and slow) decay model for our analysis, we saw minimal differences in rate of tetramer dissociation in pre-vaccinated and post-vaccinated samples [29] (Supplementary Fig. 4), suggesting that vaccination did not lead to a change in circulating MAIT cell TCR avidity.

### Ty21a vaccination resulted in an increased expression of tissue-homing chemokine receptors and integrin on MAIT cells

**Figure 4.**
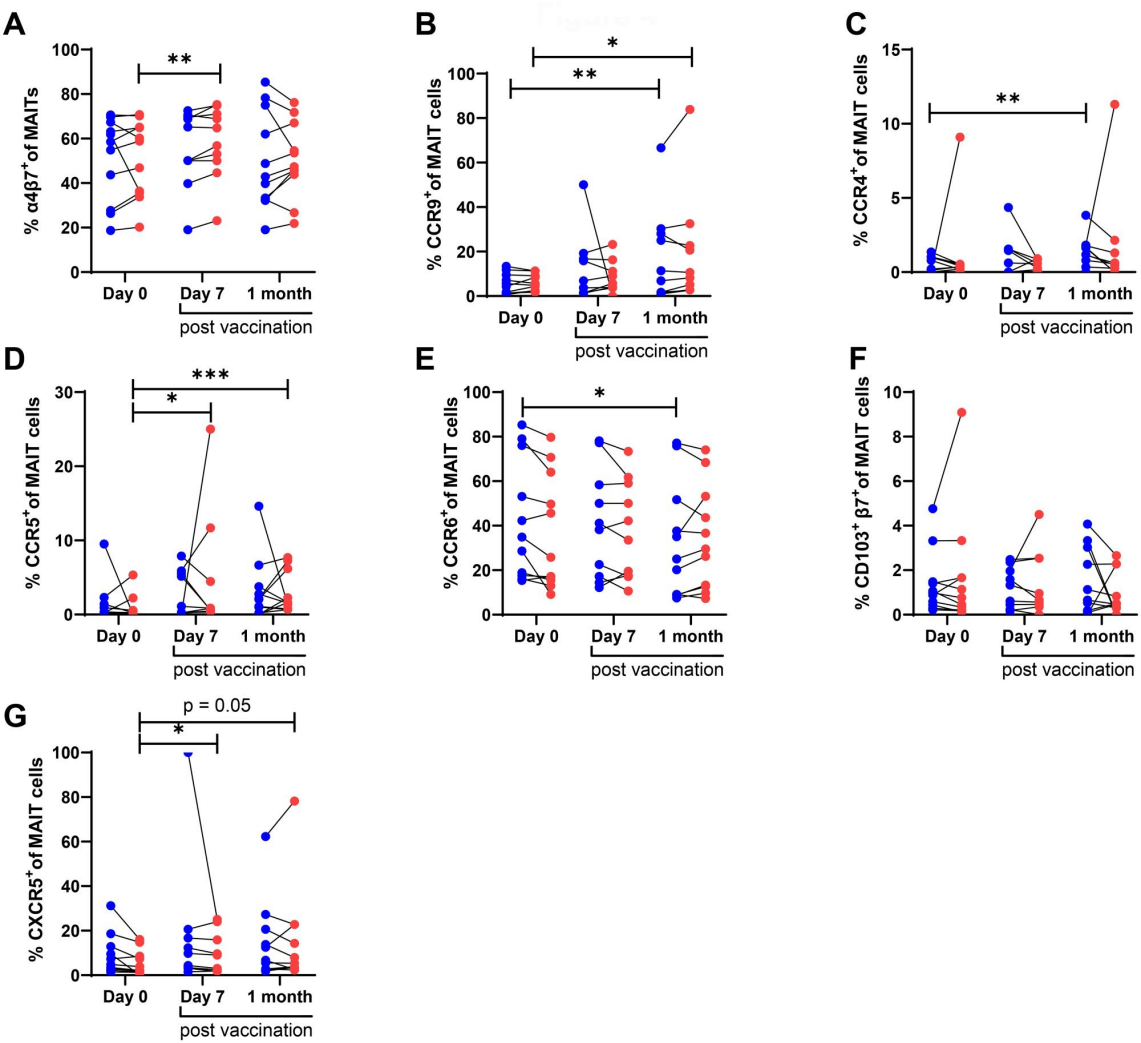
Increased homing markers and chemokine receptor expression in MAIT cells post vaccination. PBMCs obtained from vaccine recipients (*n* = 11 per group) were stimulated with heat-killed *S.* Typhi at MOI of 100 and surface expression of (A) Integrin α4β7, (B) CCR9, (C) CD103, (D) CCR4, (E) CCR5, (F) CCR6, (G) CXCR5 expression in MAIT cells was measured using flow cytometry. Data were expressed as mean ± SEM. The data are representative of two independent experiments out of a total of two. Paired comparisons were made using Wilcoxon matched-pairs signed-rank test.

Given that we found lower frequencies of circulating MAIT cells post-vaccination, it is possible that MAIT cells home to other compartments, such as the gut or other mucosal tissues. To address this possibility, we measured the expression of integrin α4β7 and CCR9 molecules, gut-homing markers known to be found on MAIT cells [30–33]. We found that the proportion of MAIT cells expressing integrin α4β7 increases at day 7 post-vaccination upon stimulation (Fig. 4A and Supplementary Figs 3 and 5), and the proportion of MAIT cells expressing CCR9, were higher one-month post-vaccination in unstimulated and stimulated conditions (Fig. 4B). We found similar results when we analyzed integrin α4β7 and CCR9 molecules in Vα7.2^+^ CD161+ population and when we analyzed median fluorescent intensity (MFI) of integrin α4 and integrin β7 (Supplementary Fig. 5). Next, we used a trans-well chemotaxis assay to assess the potential migration of MAIT cells in the presence of CCR9 ligand CCL25/TECK-3. We found an observed increase in MAIT cell migration in response to CCL25 at one-month post-vaccination compared to pre-vaccination, although this analysis was limited by the insufficient PBMC sample size at this later time point (*P* = 0.25, Supplementary Fig. 6). It is important to note that this increase was not statistically significant. We also found higher percentages of MAIT cells expressing CCR4, CCR5 and CCR6 chemokine receptors, which are involved in tissue homing [12, 34–36], one-month post-vaccination (Fig. 4C–E). No significant differences were observed in MAIT cells expressing CD103/αE β7 integrin (skin/liver homing [30]) expression (Fig. 4F) and MAIT cell chemotaxis for CCR6 ligand MIP-3α (Supplementary Fig. 6). Furthermore, given recent identification of CXCR5^+^ follicular helper-like MAIT cells [22], we found a higher proportion of stimulated MAIT cells at post-vaccination time points to be positive for CXCR5^+^ (Fig. 4G). Taken together, we found evidence that the decreased proportion of MAIT cells in blood may be associated with increased homing and migration into tissues following Ty21a vaccination.

## Discussion

MAIT cells have been implicated in protective responses to microbial infections and, due to their abundance in humans, capacity for rapid effector functions, and lack of restriction by donor genotype, could be attractive vaccine targets [14, 15]. While previous studies have described MAIT cell response to systemic vaccinations in humans [11, 37], mice [8] and macaques [38], there are very limited data on how MAIT cells respond to mucosal vaccines. In this longitudinal study, we evaluated MAIT cell response seven days and one-month after oral *S*. Typhi strain Ty21a vaccination, and found changes in circulating MAIT cell frequency, activation and homing markers.

We found that circulating MAIT cells are reduced in frequency and activated following Ty21a vaccination. In humans, similar reductions in MAIT cell frequencies have been observed in the blood of children following *Vibrio cholerae* infection [7], patients with active *Mycobacterium tuberculosis* [39], and people living with human immunodeficiency virus (HIV) infection [40]. Our results are in line with a prior study demonstrating that oral challenge of participants with wild-type *S.* Typhi results in a sharp decline of circulating MAIT cells 48 and 96 h after typhoid diagnosis, with MAIT cells highly activated and co-expressing CCR6 and CCR9 homing markers [10]. Similarly, our results are also consistent with a study of subjects receiving an attenuated *Shigella* vaccine, which also demonstrated alterations in MAIT cell frequency and activation [11]. One possible explanation for the lower frequencies of MAIT cells in circulation post-vaccination is their migration to mucosal tissues, where they may be recruited to contribute to innate effector responses [41]. In support of this, we found that MAIT cells post-vaccination had increased expression of several mucosal-homing chemokine receptors, including CCR6, CCR9 and integrin α4β7. However, we did not measure the expression of chemokines like CCL25 (for CCR9) or adhesion molecules like MAdCAM-1 (for α4β7 binding) in the gut, and in the case of insufficient homing signals, MAIT cells might fail to enter the tissue, leading to the retention of α4β7^+^ or CCR9^+^ MAIT cells in circulation. Further work is needed to determine the contribution and activity of MAIT cells homing to the gut post-Ty21a vaccination.

MAIT cells produce effector molecules such as IFN-γ, TNF and cytotoxic molecules required for killing and eliminating bacteria-infected cells. In our study, we found increased frequencies of TNF expressing circulating MAIT cells post-vaccination but no significant differences in cytotoxic molecules. This highlights the context-dependent functional differences of MAIT cells, as tissue-resident MAIT cells can have different phenotypes and cytokine production than those in the circulation [42, 43]. We also found that while pre-vaccination samples showed significant increases in TNF-α, IFN-γ, and perforin upon ex-vivo stimulation with heat-killed *S*. Typhi, this was not observed in post-vaccination samples. We used heat-killed *S*. Typhi to mimic the immune responses of MAIT cells *in vivo*. Notably, post-vaccination MAIT cells, in the absence of stimulation, had a higher level of activation marker CD38, and correspondingly, higher levels of expression of cytokines (TNF in particular). The reason for this phenomenon is unclear and could partly be due to a non-antigen-driven activation of MAIT cells that persists following mucosal vaccination. According to Boulouis *et al*. [44], pre-vaccination and post-vaccination levels of MAIT cells correlated positively with the magnitude of the immune response to the BNT162b2 mRNA SARS-CoV-2 vaccine indicating non-antigen driven activation of MAIT cells. The Ty21a vaccination might have primed the immune system in a way that enhances the cytokine response, leading to a baseline activation state of MAIT cells and resulting in higher expression levels of the activation markers.

Our study has a number of limitations. First, due to the preliminary nature of this study, we had a small sample size, although this did not preclude us in observing significant changes in MAIT cell response post-Ty21a vaccination. Studies with larger cohorts are required to confirm and expand our findings. Second, our study was limited to the description of MAIT cells in blood. Given our findings of increased homing markers and migration, future studies examining tissue-resident MAIT cells are needed. Third, the overall MAIT cytokine response was low upon *ex-vivo* stimulation with heat-killed *S.* Typhi, likely due to the heat sensitivity of MAIT cell-activating antigens, such as riboflavin metabolites [20, 45]. We lacked sufficient cells to evaluate MAIT effector functions with alternative known and potent stimulations (e.g. MR1 ligand 5-OP-RU or cytokines). Fourth, a limitation of our tetramer dissociation assay is the time points used for measuring MAIT cell avidity. Our current experimental design included points beyond 1 h but given the relatively fast kinetics of MAIT TCR-MR1 interactions, shorter time intervals (e.g. within 1 h) may provide a more precise assessment of avidity.

Despite these limitations, our study expands the limited body of evidence on MAIT cell responses to oral vaccination. Specifically, we provide new insights into MAIT cell dynamics in the context of *S*. Typhi vaccination: (i) Unlike previous studies focused on acute infection [9, 10], this study investigated longitudinal vaccine-induced changes in MAIT cells which may inform the development of MAIT-targeted vaccines strategies. (ii) We show that vaccination modulates MAIT cell TNF production and migration markers, suggesting that vaccines can have longer-term impact on MAIT cell responses. Given the global burden of enteric infections, there is an urgent need for more effective mucosal vaccines, particularly oral formulations [46]. Our findings provide an important step towards the understanding and harnessing of MAIT cell function, which may offer an important therapeutic strategy to improve mucosal immunity.

## Supplementary Material

iqaf002_Supplementary_Data

## Data Availability

All data generated or analyzed during this study are included in the manuscript and supporting files.

## References

[1] Lepore M , Kalinichenko A, Colone A et al Parallel T-cell cloning and deep sequencing of human MAIT cells reveal stable oligoclonal TCRbeta repertoire. Nat Commun 2014;5:3866. 10.1038/ncomms4866 24832684

[2] Reantragoon R , Corbett AJ, Sakala IG et al Antigen-loaded MR1 tetramers define T cell receptor heterogeneity in mucosal-associated invariant T cells. J Exp Med 2013;210:2305–20. 10.1084/jem.20130958 24101382 PMC3804952

[3] Kjer-Nielsen L , Patel O, Corbett AJ et al MR1 presents microbial vitamin B metabolites to MAIT cells. Nature 2012;491:717–23. 10.1038/nature11605 23051753

[4] Meierovics A , Yankelevich WJ, Cowley SC. MAIT cells are critical for optimal mucosal immune responses during in vivo pulmonary bacterial infection. Proc Natl Acad Sci U S A 2013;110:E3119–28. 10.1073/pnas.1302799110 23898209 PMC3746930

[5] Chua WJ , Truscott SM, Eickhoff CS et al Polyclonal mucosa-associated invariant T cells have unique innate functions in bacterial infection. Infect Immun 2012;80:3256–67. 10.1128/IAI.00279-12 22778103 PMC3418730

[6] Georgel P , Radosavljevic M, Macquin C, Bahram S. The non-conventional MHC class I MR1 molecule controls infection by Klebsiella pneumoniae in mice. Mol Immunol 2011;48:769–75. 10.1016/j.molimm.2010.12.002 21190736

[7] Leung DT , Bhuiyan TR, Nishat NS et al Circulating mucosal associated invariant T cells are activated in Vibrio cholerae O1 infection and associated with lipopolysaccharide antibody responses. PLoS Negl Trop Dis 2014;8:e3076. 10.1371/journal.pntd.0003076 25144724 PMC4140671

[8] Wang H , D'Souza C, Lim XY et al MAIT cells protect against pulmonary Legionella longbeachae infection. Nat Commun 2018;9:3350. 10.1038/s41467-018-05202-8 30135490 PMC6105587

[9] Howson LJ , Napolitani G, Shepherd D et al MAIT cell clonal expansion and TCR repertoire shaping in human volunteers challenged with Salmonella Paratyphi A. Nat Commun 2018;9:253. 10.1038/s41467-017-02540-x 29343684 PMC5772558

[10] Salerno-Goncalves R , Luo D, Fresnay S et al Challenge of humans with wild-type Salmonella enterica Serovar Typhi elicits changes in the activation and homing characteristics of mucosal-associated invariant T cells. Front Immunol 2017;8:398. 10.3389/fimmu.2017.00398 28428786 PMC5382150

[11] Le Bourhis L , Dusseaux M, Bohineust A et al MAIT cells detect and efficiently lyse bacterially-infected epithelial cells. PLoS Pathog 2013;9:e1003681. 10.1371/journal.ppat.1003681 24130485 PMC3795036

[12] Dusseaux M , Martin E, Serriari N et al Human MAIT cells are xenobiotic-resistant, tissue-targeted, CD161hi IL-17-secreting T cells. Blood 2011;117:1250–9. 10.1182/blood-2010-08-303339 21084709

[13] Le Bourhis L , Martin E, Peguillet I et al Antimicrobial activity of mucosal-associated invariant T cells. Nat Immunol 2010;11:701–8. 10.1038/ni.1890 20581831

[14] Downey AM , Kapłonek P, Seeberger PH. MAIT cells as attractive vaccine targets. FEBS Lett 2019;593:1627–40. 10.1002/1873-3468.13488 31206659

[15] Salou M , Franciszkiewicz K, Lantz O. MAIT cells in infectious diseases. Curr Opin Immunol 2017;48:7–14. 10.1016/j.coi.2017.07.009 28750261

[16] Boulouis C , Sia WR, Gulam MY et al Human MAIT cell cytolytic effector proteins synergize to overcome carbapenem resistance in Escherichia coli. PLoS Biol 2020;18:e3000644. 10.1371/journal.pbio.3000644 32511236 PMC7302869

[17] Provine NM , Amini A, Garner LC et al MAIT cell activation augments adenovirus vector vaccine immunogenicity. Science 2021;371:521–6. 10.1126/science.aax8819 33510029 PMC7610941

[18] Crump JA , Luby SP, Mintz ED. The global burden of typhoid fever. Bull World Health Organ 2004;82:346–53. 15298225 PMC2622843

[19] Pennington SH , Ferreira DM, Caamano-Gutierrez E et al Nonspecific effects of oral vaccination with live-attenuated Salmonella Typhi strain Ty21a. Sci Adv 2019;5:eaau6849. 10.1126/sciadv.aau6849 30820452 PMC6392763

[20] Dias J , Sobkowiak MJ, Sandberg JK, Leeansyah E. Human MAIT-cell responses to Escherichia coli: activation, cytokine production, proliferation, and cytotoxicity. J Leukoc Biol 2016;100:233–40. 10.1189/jlb.4TA0815-391RR 27034405 PMC4946616

[21] Bennett MS , Trivedi S, Iyer AS et al Human mucosal-associated invariant T (MAIT) cells possess capacity for B cell help. J Leukoc Biol 2017;102:1261–9. 10.1189/jlb.4A0317-116R 28807929 PMC5636046

[22] Jensen O , Trivedi S, Meier JD et al A subset of follicular helper-like MAIT cells can provide B cell help and support antibody production in the mucosa. Sci Immunol 2022;7:eabe8931. 10.1126/sciimmunol.abe8931 35030034 PMC9001248

[23] Ibidapo-Obe O , Stengel S, Kose-Vogel N et al Mucosal-associated invariant T cells redistribute to the peritoneal cavity during spontaneous bacterial peritonitis and contribute to peritoneal inflammation. Cell Mol Gastroenterol Hepatol 2020;9:661–77. 10.1016/j.jcmgh.2020.01.003 31954178 PMC7160599

[24] Andrews JR , Khanam F, Rahman N et al Plasma immunoglobulin A responses against 2 Salmonella Typhi antigens identify patients with typhoid fever. Clin Infect Dis 2019;68:949–55. 10.1093/cid/ciy578 30020426 PMC6399438

[25] Bartholomeusz RC , Forrest BD, Labrooy JT et al The serum polymeric IgA antibody response to typhoid vaccination; its relationship to the intestinal IgA response. Immunology 1990;69:190–4. 2307480 PMC1385588

[26] D'Amelio R , Tagliabue A, Nencioni L et al Comparative analysis of immunological responses to oral (Ty21a) and parenteral (TAB) typhoid vaccines. Infect Immun 1988;56:2731–5. 10.1128/iai.56.10.2731-2735.1988 3417354 PMC259636

[27] Viret JF , Favre D, Wegmuller B et al Mucosal and systemic immune responses in humans after primary and booster immunizations with orally administered invasive and noninvasive live attenuated bacteria. Infect Immun 1999;67:3680–5. 10.1128/IAI.67.7.3680-3685.1999 10377160 PMC116565

[28] Lundin BS , Johansson C, Svennerholm AM. Oral immunization with a Salmonella enterica serovar typhi vaccine induces specific circulating mucosa-homing CD4(+) and CD8(+) T cells in humans. Infect Immun 2002;70:5622–7. 10.1128/IAI.70.10.5622-5627.2002 12228290 PMC128315

[29] Souter MNT , Awad W, Li S et al CD8 coreceptor engagement of MR1 enhances antigen responsiveness by human MAIT and other MR1-reactive T cells. J Exp Med 2022;219:e20210828. 10.1084/jem.20210828 PMC9424912 36018322

[30] Jeffery HC , van Wilgenburg B, Kurioka A et al Biliary epithelium and liver B cells exposed to bacteria activate intrahepatic MAIT cells through MR1. J Hepatol 2016;64:1118–27. 10.1016/j.jhep.2015.12.017 26743076 PMC4822535

[31] Maleki KT , Tauriainen J, Garcia M et al MAIT cell activation is associated with disease severity markers in acute hantavirus infection. Cell Rep Med 2021;2:100220. 10.1016/j.xcrm.2021.100220 33763658 PMC7974553

[32] Juno JA , Wragg KM, Amarasena T et al MAIT cells upregulate alpha4beta7 in response to acute simian immunodeficiency virus/simian HIV infection but are resistant to peripheral depletion in pigtail macaques. J Immunol 2019;202:2105–20. 10.4049/jimmunol.1801405 30777923

[33] Sundstrom P , Ahlmanner F, Akeus P et al Human mucosa-associated invariant T cells accumulate in colon adenocarcinomas but produce reduced amounts of IFN-gamma. J Immunol 2015;195:3472–81. 10.4049/jimmunol.1500258 26297765

[34] Billerbeck E , Kang YH, Walker L et al Analysis of CD161 expression on human CD8+ T cells defines a distinct functional subset with tissue-homing properties. Proc Natl Acad Sci U S A 2010;107:3006–11. 10.1073/pnas.0914839107 20133607 PMC2840308

[35] Hanson ED , Danson E, Evans WS et al Exercise increases mucosal-associated invariant T cell cytokine expression but not activation or homing markers. Med Sci Sports Exerc 2019;51:379–88. 10.1249/MSS.0000000000001780 30649094 PMC6424664

[36] Wong EB , Akilimali NA, Govender P et al Low levels of peripheral CD161++CD8+ mucosal associated invariant T (MAIT) cells are found in HIV and HIV/TB Co-infection. Plos ONE 2013;8:e83474. 10.1371/journal.pone.0083474 24391773 PMC3877057

[37] Gela A , Murphy M, Rodo M, Delayed BCG Study Team et al Effects of BCG vaccination on donor unrestricted T cells in two prospective cohort studies. EBioMedicine 2022;76:103839. 10.1016/j.ebiom.2022.103839 35149285 PMC8842032

[38] Greene JM , Dash P, Roy S et al MR1-restricted mucosal-associated invariant T (MAIT) cells respond to mycobacterial vaccination and infection in nonhuman primates. Mucosal Immunol 2017;10:802–13. 10.1038/mi.2016.91 27759023 PMC5397382

[39] Gold MC , Cerri S, Smyk-Pearson S et al Human mucosal associated invariant T cells detect bacterially infected cells. PLoS Biol 2010;8:e1000407. 10.1371/journal.pbio.1000407 20613858 PMC2893946

[40] Leeansyah E , Ganesh A, Quigley MF et al Activation, exhaustion, and persistent decline of the antimicrobial MR1-restricted MAIT-cell population in chronic HIV-1 infection. Blood 2013;121:1124–35. 10.1182/blood-2012-07-445429 23243281 PMC3575756

[41] Voillet V , Buggert M, Slichter CK et al Human MAIT cells exit peripheral tissues and recirculate via lymph in steady state conditions. JCI Insight 2018;3:e98487. 10.1172/jci.insight.98487 29618662 PMC5928862

[42] Amini A , Pang D, Hackstein CP, Klenerman P. MAIT cells in barrier tissues: lessons from immediate neighbors. Front Immunol 2020;11:584521. 10.3389/fimmu.2020.584521 33329559 PMC7734211

[43] Bhuiyan TR , Rahman MA, Trivedi S et al Mucosal-associated invariant T (MAIT) cells are highly activated in duodenal tissue of humans with Vibrio cholerae O1 infection: A preliminary report. PLoS Negl Trop Dis 2022;16:e0010411. 10.1371/journal.pntd.0010411 35551522 PMC9129025

[44] Boulouis C , Kammann T, Cuapio A, COVAXID study group et al MAIT cell compartment characteristics are associated with the immune response magnitude to the BNT162b2 mRNA anti-SARS-CoV-2 vaccine. Mol Med 2022;28:54. 10.1186/s10020-022-00484-7 35562666 PMC9100314

[45] Salerno-Goncalves R , Rezwan T, Sztein MB. B cells modulate mucosal associated invariant T cell immune responses. Front Immunol 2014;4:511. 10.3389/fimmu.2013.00511 24432025 PMC3882667

[46] Davitt CJ , Lavelle EC. Delivery strategies to enhance oral vaccination against enteric infections. Adv Drug Deliv Rev 2015;91:52–69. 10.1016/j.addr.2015.03.007 25817337

