# Supplementary material for: Mucosal-associated invariant T (MAIT) cell responses in Salmonella enterica serovar Typhi strain Ty21a oral vaccine recipients
Source: Oxf Open Immunol. 2025 Mar 25;6(1):iqaf002. doi: 10.1093/oxfimm/iqaf002 (PMC11993846; doi:10.1093/oxfimm/iqaf002)
Supplement: iqaf002_Supplementary_Data [file iqaf002_supplementary_data.zip › Supplementary figures and table final.pdf]

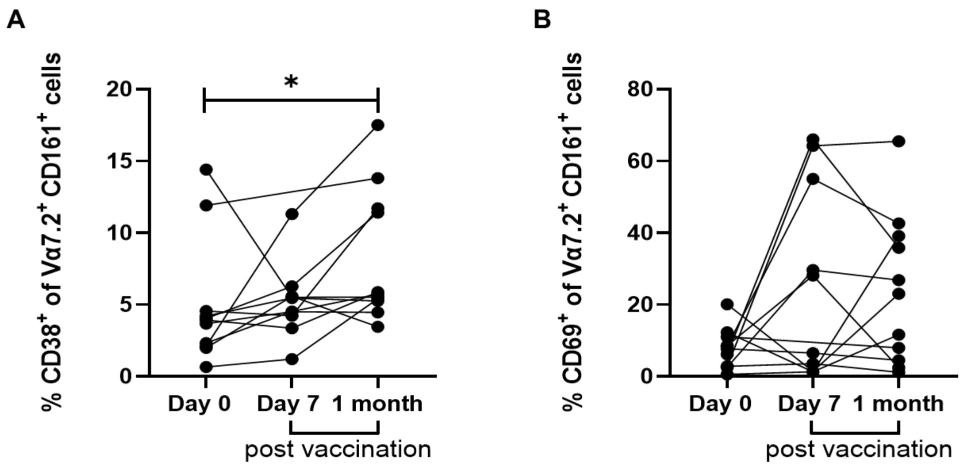


**Supplementary Figure 1. Activation marker CD38 and CD69 expression in Vα7.2$^{+}$ CD161$^{+}$ MAIT cells post vaccination.** PBMCs obtained from vaccine recipients (n = 11 per group) were stimulated with *S.* Typhi at moi of 100 and surface expression of **(A)** CD38, **(B)** CD69 in Vα7.2$^{+}$ CD161$^{+}$ MAIT cells was measured using flow cytometry. Data were expressed as mean ± SEM. The data are representative of two independent experiments out of a total of two. *$P$ < .05, **$P$ < .01, ***$P$ < .001 in Wilcoxon signed-rank test (paired samples).

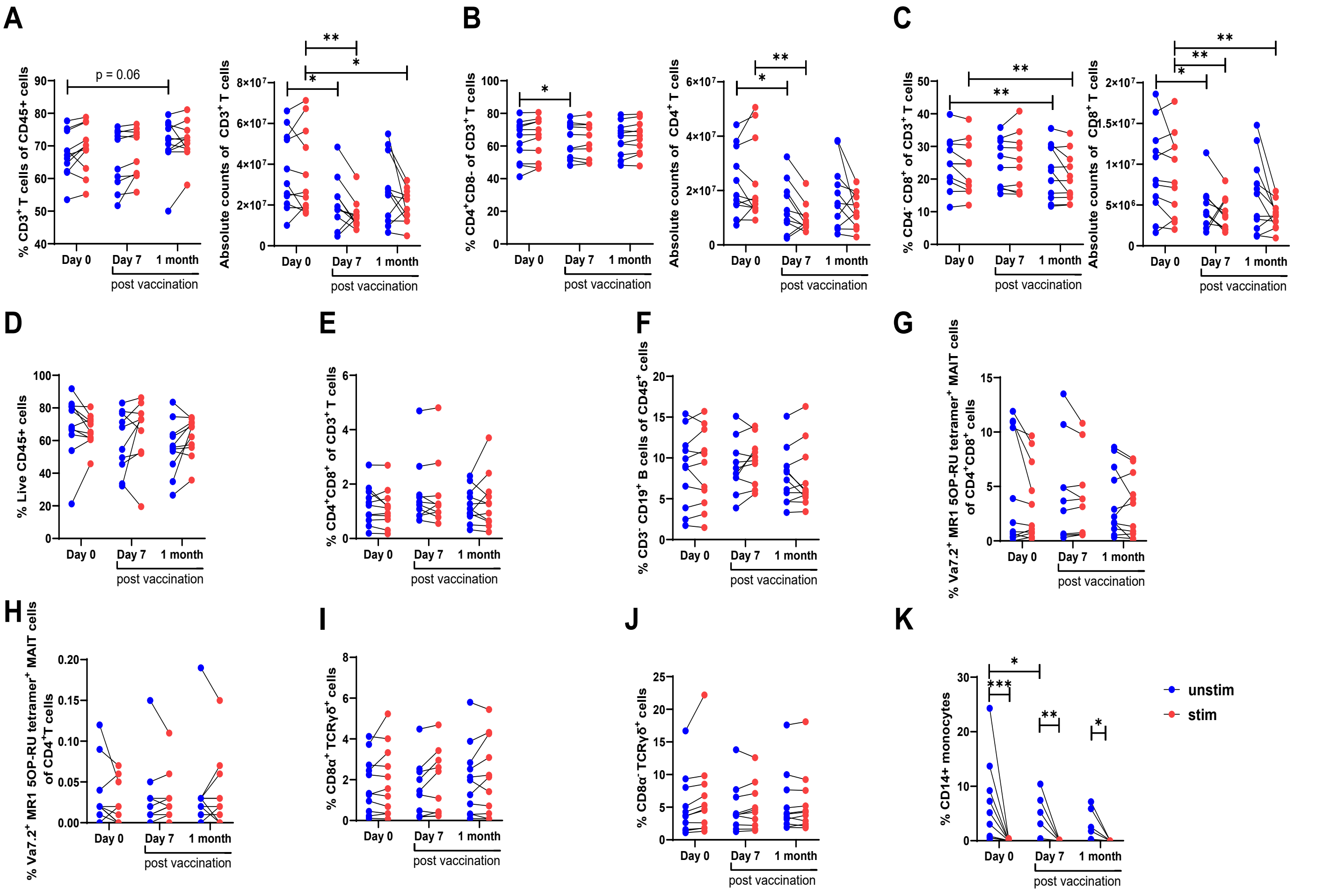


**Supplementary Figure 2. Effect of Ty21a vaccination on CD4+CD8+ MAIT cells, CD4+ MAITs and other non-MAIT populations.** Frequencies and absolute counts of **(A)** CD3+ T cells, **(B)** CD4+ T cells, **(C)** CD8+ T cells and percentage frequencies of **(D)** CD45+ cells, **(E)** CD4+ CD8+ T cells, , **(F)** CD19+ B cells, **(G)** CD4+CD8+ MAIT cells, **(H)** CD4+ MAIT cells, **(I)** CD8+ TCRγδ+ cells , **(J)** CD8- TCRγδ+ cells and **(K)** CD14+ monocytes at day 0 pre vaccination and day 7 and one month post vaccination (blue circles are unstimulated and red circles are stimulated). Data were expressed as mean ± SEM. The data are representative of two independent experiments out of a total of two. *$P$ < .05, **$P$ < .01, ***$P$ < .001 in Wilcoxon signed-rank test (paired samples).

**Representative FACS plots gated on Vα7.2+ 5-OP-RU+ CD8+ MAIT cells as shown in Figure 2A.**

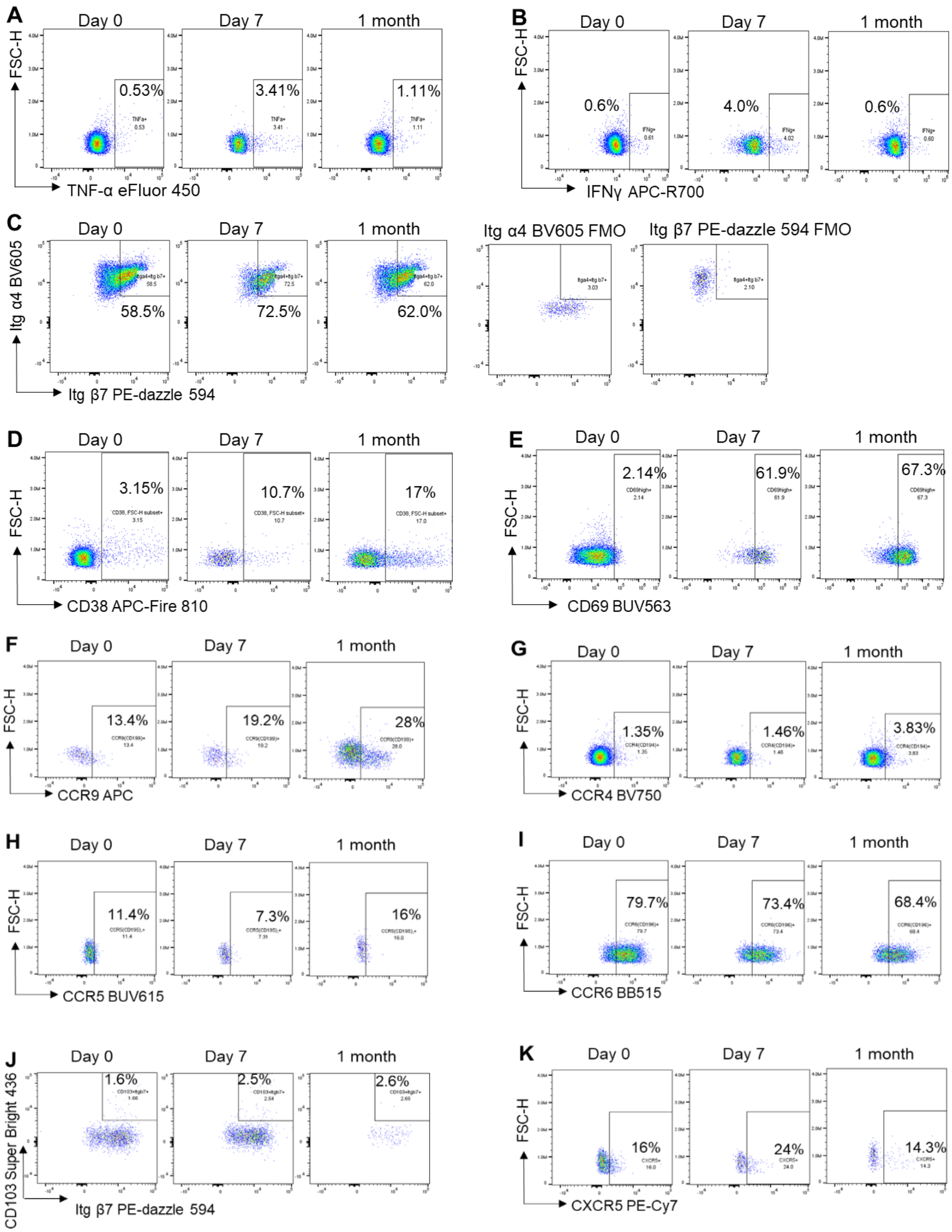


**Supplementary Figure 3. Representative flow cytometry plots of MAIT cell cytokine and homing marker expression.** PBMCs obtained at day 0, day 7- and one-month post vaccination were

stained and gated on MAIT cells as mentioned in methods and figure 2. Representative flow cytometry plots are shown at each time point for (A) TNFα, (B) IFN-γ, (C) Integrin α4β7, (D) CD38, (E) CD69, (F) CCR9, (G) CCR4, (H) CCR5, (I) CCR6, (J) Integrin CD103β7, (K) CXCR5.

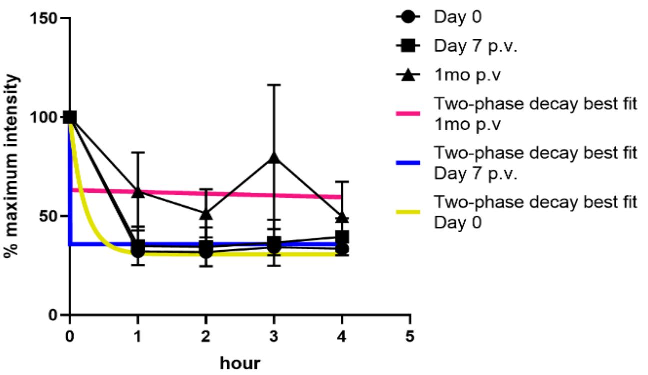


| | Day 0 | Day 7 p.v. | 1mo p.v |
|---|---|---|---|
| KFast | 4.651 | Unstable | Unstable |
| KSlow | 5.365e-009 | 1.686e-011 | 0.01464 |

**Supplementary Figure 4. MAIT cells tetramer dissociation post-vaccination.** MAIT cell (CD3+ 5-OP-RU tetramer+) tetramer decay in the presence of 20 µg/mL MR1 blocking antibody (aMR1) is plotted as percent maximum intensity pre and post vaccination. The percentage maximum intensity was calculated by normalizing the MFI (median fluorescent intensity) at each time to maximum MFI at time 0. A two phase (fast and slow) decay model was used for analysis and solid-colored lines shows best-fit, $k_{fast}$, fast rate constant; $k_{slow}$, slow rate constant. Data were expressed as mean ± SEM. The data are representative of two independent experiments out of a total of two.

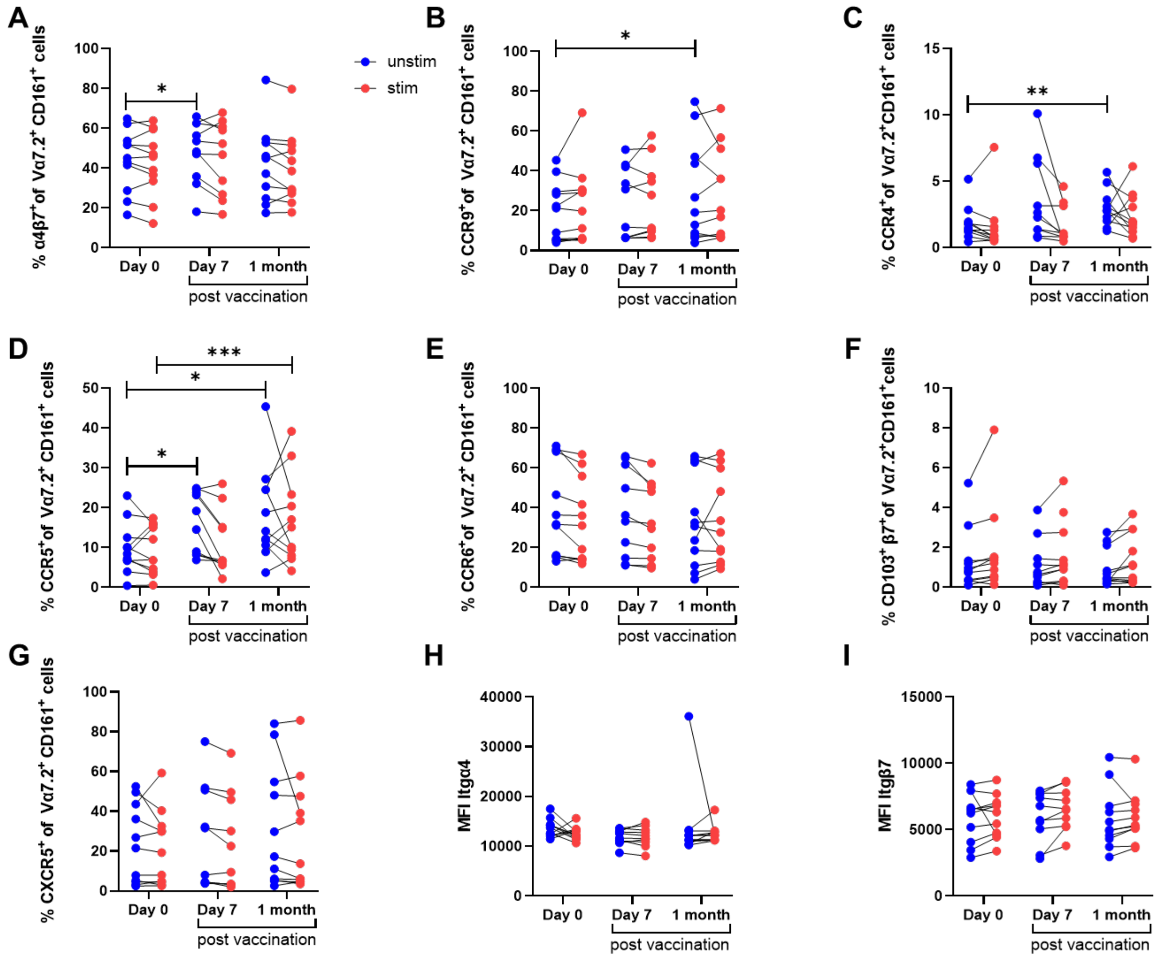


**Supplementary Figure 5. Homing markers and chemokine receptor expression in Vα7.2$^+$ CD161$^+$ MAIT cells post vaccination.** PBMCs obtained from vaccine recipients (n = 11 per group) were stimulated with *S.* Typhi at MOI of 100 and surface expression of **(A)** Integrin α4β7, **(B)** CCR9, **(C)** CCR4, **(D)** CCR5, **(E)** CCR6, **(F)** Integrin CD103β7 **(G)** CXCR5 in Vα7.2$^+$ CD161$^+$ MAIT cells and **(H and I)** Median fluorescent intensity of integrin α4 and integrin β7 in 5-OP-RU tetramer+ MAIT cells was measured using flow cytometry. Data were expressed as mean ± SEM. The data are representative of two independent experiments out of a total of two. **P* < .05, ***P* < .01, ****P* < .001 in Wilcoxon signed-rank test (paired samples).

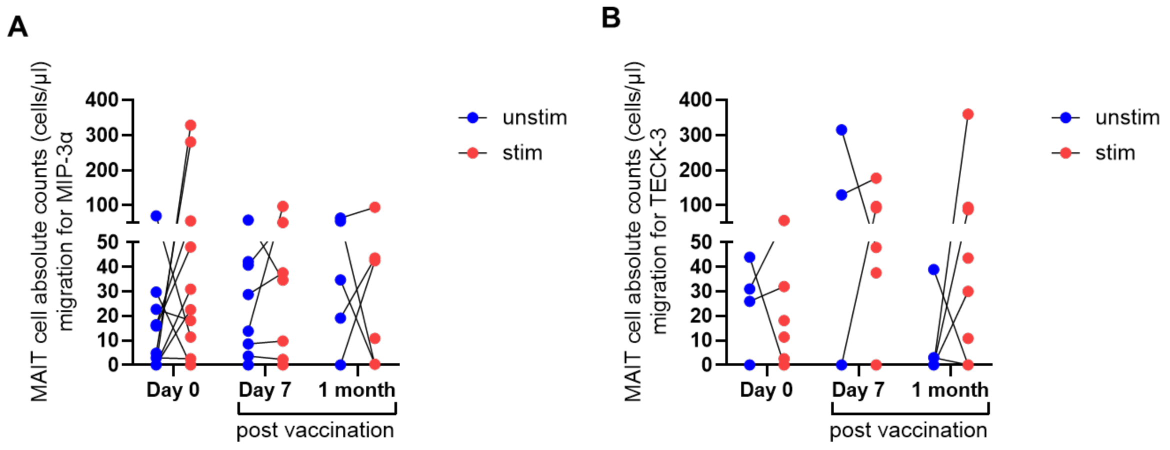


**Supplementary Figure 6. MAIT cell migration towards MIP-3α and TECK-3 chemokines.** PBMCs from vaccine recipients were seeded on upper chamber of 3-µm pore trans well with indicated chemokines at 150 ng/mL in the bottom well and were allowed to migrate for 4 hours at 37°C. Absolute numbers of MAIT cells migrated towards **(A)** MIP-3α and **(B)** TECK-3 chemokines were quantified using flow cytometry. Data were expressed as mean ± SEM. The data are representative of two independent experiments out of a total of two. *$P < .05$, **$P < .01$, ***$P < .001$ in Wilcoxon signed-rank test (paired samples).

## Supplementary table 1

| Number | Purpose | Antigen | Clone | Fluorophore | Dilution used | Catlogue # | Company |
|---|---|---|---|---|---|---|---|
| 1 | Lineage marker | CD3 | UCHT1 | BUV496 | 1 in 200 | 612940 | BD |
| 2 | Lineage marker | CD8 | RPAT8 | BV570 | 1 in 200 | 301038 | Biolegend |
| 3 | Lineage marker | CD4 | RPA-T4 | BUV737 | 1 in 200 | 741823 | BD |
| 4 | Lineage marker | CD45 | HI30 | BUV805 | 1 in 200 | 612892 | BD |
| 5 | Basic gating live dead | dead | n/a | Zombie UV | 1 in 2000 | 423107 | Biolegend |
| 6 | TCRgamma delta | TCRgd | 11f2 | BV421 | 1 in 100 | 744870 | BD |
| 7 | B cell | CD19 | HIBI9 | spark NIR685 | 1 in 100 | 302270 | Biolegend |
| 8 | Monocytes | CD14 | 63D3 | spark blue 550 | 1 in 100 | 367148 | Biolegend |
| 9 | MAIT cell marker | MR1 tetramer | n/a | PE | 1 in 400 | n/a | NIH |
| 10 | MAIT cell marker | Va7.2 | OF-5A12 | BV711 | 1 in 100 | 749489 | BD |
| 11 | MAIT cell marker | CD161 | DX12 | BUV661 | 1 in 100 | 750382 | BD |
| 12 | MAIT activation | CD69 | FN 50 | BUV563 | 1 in 200 | 748764 | BD |
| 13 | MAIT activation | CD38 | HB7 | APC Fire 810 | 1 in 200 | 356643 | Biolegend |
| 14 | MAIT homing | CCR9(CD199) | L053E8 | apc | 1 in 100 | 358908 | Biolegend |
| 15 | MAIT homing | Integrin alpha 4 CD49d | 9F10 | BV605 | 1 in 100 | 304324 | Biolegend |
| 16 | MAIT homing | Integrin beta 7 | FIB504 | PE dazzle 594 | 1 in 100 | 321226 | Biolegend |
| 17 | MAIT homing | CD103 | Ber ACT8 | superbright 436 | 1 in 100 | 62-1038-42 | Thermo fisher scientific |
| 18 | MAIT chemokine recptor | CCR5 (CD195) | 3A9 | BUV615 | 1 in 100 | 751586 | BD |
| 19 | MAIT chemokine recptor | CCR4(CD194) | 1G1 | BV750 | 1 in 100 | 746980 | BD |
| 20 | MAIT chemokine recptor | CCR6 (CD196) | 11A9 | BB515 | 1 in 100 | 564479 | BD |
| 21 | MAIT function | IFNg | B27 | APC-R700 | 1 in 100 | 564981 | BD |
| 22 | MAIT function | IL-17 | N49-653 | BV650 | 1 in 100 | 563746 | BD |
| 23 | MAIT function | TNFa | MAb11 | ef450 | 1 in 100 | 48-7349-42 | Thermo fisher scientific |
| 24 | MAIT function | GzmB | QA16A02 | percp cy5.5 | 1 in 100 | 372212 | Biolegend |
| 25 | MAIT function | Perforin | dG9 | PerCP-efluor 710 | 1 in 100 | 50-161-24 | Thermo fisher scientific |

Supplementary Table 2: Absolute counts in all donors at day 1 pre vaccination and day 7 and 1 month post vaccination.

| | Day 1 (unstim) | | | | Day 1 (stim) | | | |
|---|---|---|---|---|---|---|---|---|
| **Donor #** | **CD3+** | **CD4+CD3+** | **CD8+ CD3+** | **MAIT** | **CD3+** | **CD4+CD3+** | **CD8+ CD3+** | **MAIT** |
| 1 | 3.05E+07 | 2.37E+07 | 5.33E+06 | 1.14E+06 | 1.60E+07 | 1.26E+07 | 2.89E+06 | 1.05E+06 |
| 2 | 1.87E+07 | 9.21E+06 | 7.47E+06 | 1.885E+05 | 1.86E+07 | 9.14E+06 | 7.13E+06 | 6.31E+05 |
| 3 | 6.62E+07 | 4.42E+07 | 1.64E+07 | 3.325E+05 | 7.13E+07 | 4.77E+07 | 1.77E+07 | 3.72E+05 |
| 4 | 6.06E+07 | 2.89E+07 | 1.86E+07 | 1.791E+06 | 4.82E+07 | 2.24E+07 | 1.21E+07 | 1.65E+06 |
| 5 | 5.20E+07 | 3.64E+07 | 1.29E+07 | 4.625E+05 | 5.63E+07 | 3.94E+07 | 1.39E+07 | 4.00E+05 |
| 6 | 1.00E+07 | 7.28E+06 | 1.62E+06 | 3.185E+05 | 1.99E+07 | 1.50E+07 | 3.25E+06 | 2.93E+05 |
| 7 | 5.33E+07 | 3.81E+07 | 1.09E+07 | 3.358E+06 | 6.73E+07 | 5.06E+07 | 1.21E+07 | 2.27E+06 |
| 8 | 3.77E+07 | 1.82E+07 | 1.16E+07 | 6.409E+05 | 3.49E+07 | 1.62E+07 | 1.06E+07 | 7.16E+05 |
| 9 | 2.45E+07 | 1.50E+07 | 6.04E+06 | 4.900E+05 | 2.64E+07 | 1.72E+07 | 5.09E+06 | 6.00E+05 |
| 10 | 2.06E+07 | 1.65E+07 | 2.34E+06 | 2.850E+05 | 1.68E+07 | 1.35E+07 | 2.01E+06 | 3.45E+05 |
| 11 | 2.56E+07 | 1.47E+07 | 8.04E+06 | 1.320E+04 | 2.38E+07 | 1.36E+07 | 7.72E+06 | 9.88E+03 |

| | Day 7 (unstim) | | | | Day 7 (stim) | | | |
|---|---|---|---|---|---|---|---|---|
| **Donor #** | **CD3+** | **CD4+CD3+** | **CD8+ CD3+** | **MAIT** | **CD3+** | **CD4+CD3+** | **CD8+ CD3+** | **MAIT** |
| 1 | 2.41E+07 | 1.95E+07 | 3.82E+06 | 3.65E+05 | 1.11E+07 | 8.87E+06 | 1.96E+06 | 4.12E+05 |
| 2 | 4.68E+06 | 2.48E+06 | 1.67E+06 | 2.57E+05 | 1.36E+07 | 7.02E+06 | 5.58E+06 | 4.01E+05 |
| 3 | 4.84E+07 | 3.24E+07 | 1.14E+07 | 9.45E+04 | 3.39E+07 | 2.26E+07 | 7.98E+06 | 4.50E+05 |
| 4 | 1.77E+07 | 9.07E+06 | 5.25E+06 | 2.24E+05 | 1.45E+07 | 7.16E+06 | 4.27E+06 | 5.43E+05 |
| 5 | 1.77E+07 | 1.27E+07 | 2.73E+06 | 1.45E+05 | 1.52E+07 | 1.08E+07 | 2.34E+06 | 1.98E+05 |
| 6 | 2.43E+07 | 1.81E+07 | 4.13E+06 | 2.45E+05 | 1.03E+07 | 7.49E+06 | 1.66E+06 | 2.45E+05 |
| 7 | 3.37E+07 | 2.46E+07 | 5.94E+06 | 1.32E+06 | 2.04E+07 | 1.50E+07 | 3.74E+06 | 1.33E+06 |
| 8 | 6.59E+06 | 3.17E+06 | 2138600 | 3.70E+05 | 1.67E+07 | 8.21E+06 | 5.76E+06 | 3.92E+05 |
| 9 | 1.42E+07 | 8.24E+06 | 3.82E+06 | 1.12E+05 | 7.90E+06 | 4.81E+06 | 2.06E+06 | 7.44E+04 |
| 10 | n/a | n/a | n/a | n/a | n/a | n/a | n/a | n/a |
| 11 | 1.92E+07 | 1.12E+07 | 6.08E+06 | 7.13E+04 | 1.13E+07 | 6.57E+06 | 3.51E+06 | 5.90E+04 |

| | 1 month (unstim) | | | | 1 month (stim) | | | |
|---|---|---|---|---|---|---|---|---|
| **Donor #** | **CD3+** | **CD4+CD3+** | **CD8+ CD3+** | **MAIT** | **CD3+** | **CD4+CD3+** | **CD8+ CD3+** | **MAIT** |
| 1 | 4.70E+07 | 3.84E+07 | 6.97E+06 | 1.15E+06 | 2.86E+07 | 2.32E+07 | 4.59E+06 | 1.16E+06 |
| 2 | 2.63E+07 | 1.44E+07 | 9.31E+06 | 2.73E+05 | 1.21E+07 | 6.76E+06 | 4.12E+06 | 7.21E+05 |
| 3 | 2.82E+07 | 1.91E+07 | 6.39E+06 | 3.57E+04 | 1.49E+07 | 1.04E+07 | 2.28E+06 | 5.46E+04 |
| 4 | 4.97E+07 | 2.52E+07 | 1.48E+07 | 7.21E+05 | 3.21E+07 | 1.76E+07 | 6.69E+06 | 1.23E+06 |
| 5 | 5.49E+07 | 3.81E+07 | 1.29E+07 | 3.11E+05 | 1.54E+07 | 1.06E+07 | 3.65E+06 | 4.79E+05 |
| 6 | 9.71E+06 | 6.44E+06 | 1.18E+06 | 7.25E+05 | 1.79E+07 | 1.19E+07 | 2.19E+06 | 1.40E+05 |
| 7 | 1.47E+07 | 1.05E+07 | 2.12E+06 | 5.54E+05 | 2.67E+07 | 1.96E+07 | 3.84E+06 | 5.11E+05 |
| 8 | 1.22E+07 | 5.86E+06 | 3.61E+06 | 4.54E+05 | 1.21E+07 | 5.78E+06 | 3.64E+06 | 4.44E+05 |
| 9 | 6.56E+06 | 4.02E+06 | 1.28E+06 | 3.43E+05 | 4.88E+06 | 2.94E+06 | 9.52E+05 | 2.47E+05 |
| 10 | 2.78E+07 | 2.21E+07 | 3.27E+06 | 4.83E+04 | 2.46E+07 | 1.96E+07 | 2.99E+06 | 7.25E+04 |
| 11 | 2.49E+07 | 1.53E+07 | 7.61E+06 | 3.61E+04 | 2.28E+07 | 1.46E+07 | 5.96E+06 | 5.51E+04 |
